# Developing Educational Animations on HIV Pre-exposure Prophylaxis (PrEP) for Women: Qualitative Study

**DOI:** 10.2196/33978

**Published:** 2022-07-08

**Authors:** Anna Marie Young, Timothee Fruhauf, Obianuju Okonkwo, Erin Gingher, Jenell Coleman

**Affiliations:** 1 Department of Gynecology and Obstetrics Johns Hopkins University School of Medicine Baltimore, MD United States

**Keywords:** PrEP, animations, education, HIV, prevention, women

## Abstract

**Background:**

Despite experiencing the second-highest rate of HIV incidence in the United States, pre-exposure prophylaxis (PrEP) use remains low among Black women due, in part, to a lack of patients’ awareness and providers’ knowledge.

**Objective:**

Our goal was to design animated educational tools informed by patients and women’s health providers to address these barriers, specifically for women at risk for HIV.

**Methods:**

Two animation storyboards about PrEP for women were created by academic stakeholders (eg, HIV clinical experts, educators, and HIV peer counselors), one for patients and one for providers. Four focus groups with community members from Baltimore, Maryland and four with women’s health providers (eg, obstetrician/gynecologists, midwives, nurse practitioners, and peer counselors) at an academic center were conducted to discuss the storyboards. Transcripts were analyzed using conventional content analysis, and themes were incorporated into the final versions of the animations.

**Results:**

Academic stakeholders and 30 focus group participants (n=16 female community members and n=14 women’s health providers) described important themes regarding PrEP. The themes most commonly discussed about the patient animation were understandability of side effects, HIV risk factors, messaging, PrEP access, and use confidence. Provider animation themes were indications for PrEP, side effects, and prescribing confidence.

**Conclusions:**

We created two PrEP animations focused on women. Stakeholder feedback highlighted the importance of ensuring the understandability and applicability of PrEP educational materials while including necessary information to facilitate use or prescribing confidence. Both community members and women’s health providers reported greater use confidence after viewing the animations.

## Introduction

Black women in the United States experience the second-highest incidence of HIV behind men who have sex with men and account for over half of new HIV diagnoses among women [1]. Many population-level factors contribute to this risk, including social and economic inequities that influence sexual networks, stigma, discrimination, and inadequate access to HIV care [2,3].

Oral HIV pre-exposure prophylaxis (PrEP) with antiretroviral drugs emtricitabine and tenofovir is a Food and Drug Administration–approved medication that prevents HIV in women up to 90% when taken daily [4-6]. The Centers for Disease Control and Prevention recommends that all sexually active adolescents and adults be informed about PrEP [7]. Furthermore, the American College of Obstetrics and Gynecology recommends PrEP for women who are at substantial risk of acquiring HIV, including those who have an HIV-positive or unknown status sexual partner, a recent sexually transmitted infection, a high number of sexual partners, report inconsistent or no condom use, participate in commercial sex work, live in a high HIV prevalent area, or inject drugs [4].

Despite the availability of PrEP, uptake has been poor among women at high risk [8,9]. Patient-level barriers to PrEP include low self-perceived risk of HIV, limited knowledge, and high perceived cost [10,11]. Provider-level barriers include poor support and infrastructure to provide PrEP, inadequate education, and underestimating patients’ risk [10,11]. Additionally, few PrEP campaigns specifically target women or women’s health providers [10,11].

Computer-based interventions, such as animations, have been associated with decreased high-risk behaviors leading to HIV acquisition [12]. Furthermore, creating multimedia tools for health education with stakeholder involvement has been encouraged to identify specific community needs and ensure effective dissemination [13,14]. Additionally, animations can decrease cognitive overload and increase attention retention and long-term recall [14,15]. Therefore, to address commonly cited barriers to PrEP uptake among women, we sought to create two women-centered PrEP animations, one for providers and one for patients, grounded in Mayer’s [15] Cognitive Theory of Multimedia Learning that posits combined auditory text and visual pictures deepens understanding more than either alone. Below, we describe the animation development process with the participation of community members and women’s health providers from Baltimore, Maryland.

## Methods

**Figure 1:**
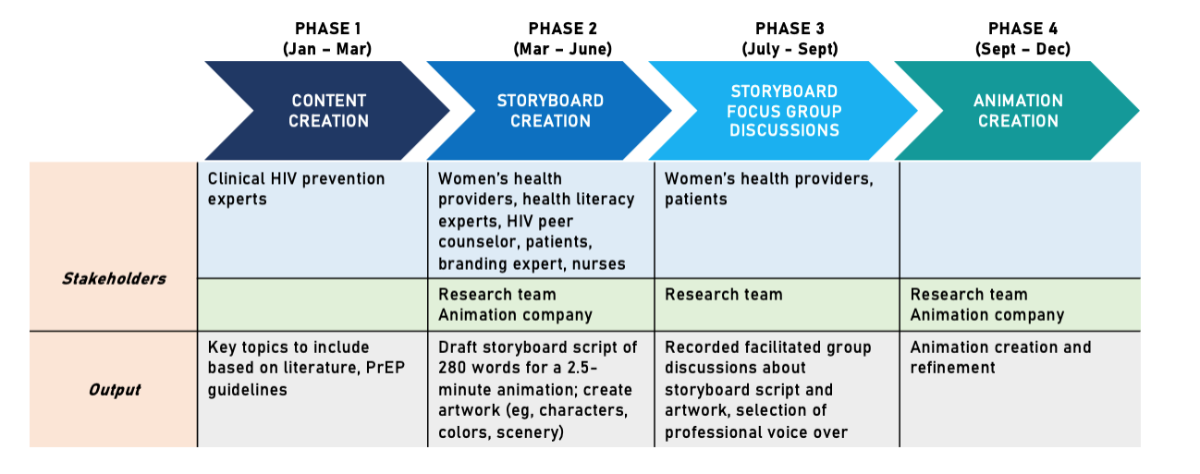
Study phases. PrEP: pre-exposure prophylaxis.

Animations were created and iteratively refined in four phases between January 2020 and December 2020 (Figure 1).

### Ethics Approval

This study was approved by the Johns Hopkins University School of Medicine Institutional Review Board (approval number: IRB00252170).

### Phase 1: Animation Content Creation

Clinical HIV prevention experts (eg, obstetrician/gynecologists with fellowship training in HIV care and HIV peer counselors) created outlines for the animations based on published literature and existing animations about PrEP [11,16-18]. Proposed content included background information about HIV and PrEP among women, risk factors for HIV, indications for PrEP, side effects of PrEP, and a step-by-step guide to taking PrEP or prescribing PrEP.

### Phase 2: Storyboard Creation

Two storyboards (ie, scripts and 2D slides) were created by an animation company (Science Animated, Cotswolds, United Kingdom) based on the outlines written in phase 1. The patient storyboard used simple language to aid low–health literacy populations, had relatable characters, and used positive framing. The provider storyboard assumed prior knowledge of women’s sexual health and was framed for practicality using medical terminology. In addition, we held formative discussions with our academic stakeholders (ie, lay individuals, a patient education professional, a branding director, nurses, and clinical HIV prevention experts), and storyboards were revised based on these initial discussions.

### Phase 3: Focus Groups

Feedback about the storyboards from a larger audience was gathered. English-speaking women from the community and women’s health providers (eg, obstetrician/gynecologists, midwives, nurse practitioners, and peer counselors) were recruited for focus groups. Fliers were placed in all general gynecology clinics, all academic specialist obstetrician/gynecologists in an academic hospital were emailed about participating, and prior research participants who had agreed to be contacted for future studies were recruited. Purposive sampling was conducted.

After obtaining written consent and collecting demographic information, the focus groups were scheduled. A trained facilitator and logistical coordinator conducted focus groups virtually using a secure videoconferencing platform (Zoom 5.5.4; Zoom Video Communications, Inc). Participants were offered the option to turn off their video and remove their names to allow partial anonymity, as all still heard voices. There were 8 focus groups, 4 for community members and 4 for women’s health providers. Each focus group contained 3 to 6 participants and lasted 60 to 90 minutes. A semistructured focus group guide was used to frame the discussions. Near the end of each focus group, participants were asked to rank 6 female-sounding professional voice-over actors who read the same script but may have had differences in inflection, cadence, pitch, or articulation. Participants received a US $25 compensatory gift card.

### Phase 4: Focus Group Analysis and Animation Creation

The focus group audio was recorded and transcribed verbatim. The transcripts were coded using ATLAS.ti 9.0.3 (ATLAS.ti Scientific Software Development GmbH), and the findings were organized and analyzed using conventional content analysis [19]. Two research team members read the transcripts twice, line-by-line, and prepared memos summarizing their preliminary findings. Next, preliminary codes were derived inductively by highlighting recurring words or statements through an iterative process. Research team members convened multiple times to discuss and compare memos, and revisit emerging themes iteratively. Discrepancies were solved by the principal investigator. Final codes were then assessed for broader concepts to generate themes through subsequent rounds of team discussion. The generation of themes was guided by an adaptation of the Model of Communication and Health Behavior Change by Kincaid [20]. The themes identified in the storyboards (phase 2) and focus group discussions were incorporated into animation prototypes. The highest-ranked voice-over options were chosen. Finally, two 120-second 2D animations were created and iteratively refined by the research team.

## Results

### Storyboard Creation (Phases 1 and 2)

Themes that were presented in the initial creation of the storyboard included accurate PrEP information, ensuring an appropriate health literacy level to reflect the target population (eg, proficient level for the provider animation and basic to below basic level for patient animation) and representative graphics/artwork (eg, multicultural characters and scenery). The scripts for the storyboards and animation graphics were refined numerous times by stakeholders.

### Focus Groups (Phase 3)

A total of 30 participants enrolled in the focus groups (n=16 female community members and n=14 providers, Table 1). Some themes pertained to both animations (messaging, design, background, side effects and risk factors, and perceptions of PrEP access and barriers), while others were only relevant to the patient animation (relatability and applicability of characters and storyline, and PrEP use confidence) or the provider animation (understandability of PrEP indications and prescribing confidence).

**Table 1:** Sociodemographic characteristics of focus group participants (N=30).

Characteristic		Community member (n=16)		Women’s health provider (n=14)
Age (years), mean (SD)		26 (6)		42 (13)
**Sex, n (%)**				
	Male	0 (0)	2 (14)	
	Female	16 (100)	12 (86)	
**Self-reported race, n (%)**				
	Black	12 (75)	3 (21)	
	Asian	0 (0)	3 (21)	
	White	1 (6)	7 (50)	
	Other	3 (19)	1 (7)	
**Highest education level, n (%)**				
	High school	7 (44)	0 (0)	
	College	6 (38)	1 (7)	
	Graduate school	2 (13)	13 (93)	
	Other	1 (6)	0 (0)	
**Marital status, n (%)**				
	Single	8 (50)	6 (43)	
	Married/union	7 (44)	7 (50)	
	Divorced	1 (6)	1 (7)	

### Provider Feedback

Providers’ most common themes included prescribing confidence, indications for PrEP, and side effects. Additional themes and respective quotes are highlighted in Multimedia Appendix 1.

#### Indications

Providers were surprised by a few of the indications for prescribing PrEP, specifically in relation to the area in which their patients were living. Several of them were surprised to learn that the HIV prevalence in a location was included in evaluating HIV risk and PrEP indications.

So, automatically living in Baltimore, it puts you at a higher risk for HIV, so really understanding that kinda stood out to me.

#### Side Effects

The level of details on side effects and their timing was a concern for some providers. Providers asked for more clarification on which side effects to expect and a given time frame for each to be included in the animation.

I wanna like know [...] how long is it [resolution of initial side-effects] gonna take? Are we talking about the next day? Are we talking about a month?

#### Prescribing Confidence

Providing a more detailed step-by-step guide applicable to the flow of a typical clinic encounter, including follow-up steps after prescribing PrEP, was recommended.

I’d first ask myself like, “Hmm, this person seems like they’re high risk for HIV.” And then, I would ask myself, “Do I – do I have testing that allows me to firmly confirm or deny the fact that they actually have HIV right now?” [...] And then, I would say to myself, like, “Okay, so I think if they like don’t have a test on file or it’s not that recent, are there any signs that I think that they’re nonetheless actively infected with HIV and I need to test them for that before, you know, starting a conversation about preventing HIV.”

Providers were, overall, confident in their ability to prescribe PrEP based on these steps but expressed doubt about their patient’s desire and ability to comply with extensive follow-up and regular lab draws.

You’re gonna have people who are not gonna wanna come in for a HIV test every three months...I mean, that’s just gonna be a deterrent for people.

### Community Member Feedback

Community members’ most common themes were understandability of side effects and risk factors, messaging, PrEP use, PrEP access, and use confidence.

#### Risk Factors

Community members were surprised by the prevalence of HIV in their community and that they themselves would qualify for PrEP based on the listed risk factors.

I would say it related to me because before then, I never knew about PrEP [...] And I think that it will be not only a big eye-opener for me but for everyone else.

I didn’t know that – um, that Baltimore City, HIV was as high as it is. They had shared it on the news, I think like a week or two ago, and liked it just kinda like caught me off-guard...

#### Side Effects

Community members were concerned about serious side effects and wanted more information about drug-drug interactions, specifically interactions between PrEP and contraceptive methods.

The stomach and the headaches, [...] that’s kinda common. But like generally, kidney and bone density, that’s not like average things.

Um, you said that it doesn’t affect pregnancy or anything like that, but is there any risk – Like, if I’m on birth control, and I supplement with PrEP, is there any effect there, or they don’t affect each other whatsoever?

#### PrEP Use

Community members highlighted confusing concepts, including PrEP’s ability to prevent HIV and the logistics of PrEP follow-up that would need to be clarified to encourage use.

How long does it last? Is it like a shot? Well, I know it’s like a pill, but like how long does it last? Like do you have to take one every day, every week, once a month?

#### Messaging

Overall, community members reported that the language and the messaging were appropriate for all education levels.

So, if we can clear it up that – Yeah, condoms alone do prevent HIV acquisition, but it’s much more effective if you use PrEP. And if you do both together that’s even better. Um, so maybe there is a way that we could kind of, like, get that message across.

#### PrEP Access

Community members thought use confidence would be impacted by information about PrEP access, especially insurance coverage. They wanted the animation to convey that it was easy to take PrEP via the step-by-step guide, which motivated participants to recommend PrEP to their peers.

I think the most surprising thing for me is that there is an option for people without insurance [...] I don’t know how true that is. Because y’all always say that, but they’ll be, like, “Yeah. There’s an option. You can take off 10 percent.” That’s not enough.

#### Use Confidence

Overall, there was a potential for greater use confidence after viewing the storyboard. Community members expressed that the storyboard motivated them to read more about PrEP and initiate a discussion with their provider.

But now, after this focus group, I’m more interested because it was kinda well-explained. I will do my own research on like the bone density and the kidneys and the side-effects, but I think after this focus group that, uh, it’s something that I will have a conversation with my doctor about.

...But, yes, I would. It’s very simple, it’s appealing. Um, if it’s 90% accurate plus on top of a condom, um, especially if you have multiple partners. Why not?

### Animation Creation (Phase 4)

Stakeholder feedback from all phases led to clarifying language modifications and additional detail to describe the background, risk factors, indications, and steps for prescribing or accessing PrEP. Specifically, the provider animation was modified to clearly delineate prescribing steps. Details about side effects and interactions were added to the patient animation. The importance of evaluating HIV risk and PrEP eligibility according to risk factors, including geographic HIV prevalence, was explained better. Finally, design changes to the characters were made to make them more relatable (Multimedia Appendixes 2 and 3).

## Discussion

### Principal Findings

Two educational animations to facilitate learning about HIV prevention and PrEP for female patients and providers were created using a user-centered approach. There were some similar themes both community members and women’s health providers wanted to highlight in the animations that included a clear demonstration about indications for PrEP, addressing barriers to PrEP use, and providing step-by-step guides to accessing or prescribing PrEP. These themes were considered the most important for both patients and providers to increase PrEP awareness and uptake among at-risk women. In addition, there was greater use confidence after viewing the storyboards.

### Comparison to Prior Work

Although we did not test the “real-world” effectiveness of the final PrEP animations in this formative study, in general, animations have been found to be effective in increasing health information recall [12,16,21]. One study used a 2 × 2 factorial design among patients with different health literacy levels to determine which features of animations improved health information recall and attitudes [21]. They found that spoken animation significantly improved recall of health information compared to written messages among low-literacy participants (*P*=.02). Additionally, there was no differences in health information recall between high- and low-literacy participants after exposure to spoken animation (*P*=.12). Furthermore, a meta-analysis demonstrated that technology-based HIV prevention interventions have been proven to be at least as efficacious as human-delivered interventions in reducing high-risk sexual behaviors [12].

### Strengths and Limitations

A notable strength of our study is the user-centered approach with key stakeholders, which has been proven to foster stronger relationships between researchers and the community [22]. This may allow greater dissemination among at-risk women and women’s health providers. However, there are limitations. First, the generalizability of these findings to other locations may be limited. We recruited providers from a single tertiary care center, who do not represent all health care providers in different settings. However, our community members reflect women most impacted by the HIV epidemic and that share similar characteristics. Second, although we collected data until we thought that theme saturation was reached, the sample of participants was small and additional themes might have been missed. Third, the animations did not provide exhaustive information and were not tested for effectiveness. However, the purpose of the animations will be to facilitate a discussion between female patients and women’s health providers as an adjunct to routine sexual health care. Additionally, we do not expect our animations to be less effective than other human-delivered interventions, as existing data has shown that technological interventions are effective [12].

### Conclusion

To increase the use of PrEP in women who live in communities with high HIV risk, the dissemination of information regarding its use in a relatable, accessible, and applicable way is vital for both patients and providers. Therefore, we included stakeholders in creating short educational PrEP animations. Stakeholders highlighted important issues to them, which included identifying individuals that qualified for PrEP, delineating key steps in accessing or prescribing PrEP, and addressing barriers to PrEP. As a result, there was greater use confidence for community members and women’s health providers after viewing the storyboards. Future research is planned to evaluate the effectiveness of the animations to increase PrEP awareness and uptake among women who are at substantial risk for HIV.

## Appendix

Multimedia Appendix 1 Supplemental Tables 1 and 2.

Multimedia Appendix 2 Provider animation.

Multimedia Appendix 3 Patient animation.

## Abbreviations

PrEP: pre-exposure prophylaxis
