# Supplementary material for: Developing Educational Animations on HIV Pre-exposure Prophylaxis (PrEP) for Women: Qualitative Study
Source: JMIR Form Res. 2022 Jul 8;6(7):e33978. doi: 10.2196/33978 (PMC9308066; doi:10.2196/33978)
Supplement: Multimedia Appendix 1 [file formative_v6i7e33978_app1.docx]

**Supplement 1:** Representative quotes from the provider and patient animations focus groups for the themes discussed

| **Provider Animation Focus Groups** |
| --- |
| **Background** |
| “Do you guys think it would be helpful to just be very specific and say among HIV negative women or like just or women at risk for HIV or something like that? […] you know, you might consider making it a little bit more explicit there.” |
| **Indications** |
| “Living in a location with high HIV prevalence. I was really impressed when I understood what that meant.” |
| “So, automatically living in Baltimore, it puts you at a higher risk for HIV, so really understanding that kinda stood out to me.” |
| “If this is supposed to be an inclusive list of all the risk factors and not just an example of risk factors, you could say something to the effect of, “Anytime you have a patient in front of you, if they fit any of these – list them – please consider starting them on PrEP,” |
| **Side Effects** |
| “I wanna like know [..] how long is it [resolution of initial side effects] gonna take? Are we talking about the next day? Are we talking about a month?” |
| **Access** |
| “So, is it this kinda thing that we have to say, “You need to contact your insurance company to see if it’s covered”? Or do we say, “It’s covered by 90 percent of insurance companies”? Or, “It is–”? You know, I don’t know. Something about that.” |
| **Adherence** |
| “I did worry about that, um, when you had that slide about, like, “Continue to use condoms, especially in the first 20 days.” It almost sounds like, “After the first 20 days –” Facilitator: [Laughs] Provider 05: “–it’s, like, not quite as important to still use – still use condoms. And you’re on this medication that will prevent HIV in – transmission in 90 percent. So, like, maybe it’s okay if you don’t, you know, like, use condoms.” You know? So, it does have a – a little bit of a mixed message there.” |
| **Prescribing Confidence** |
| “And it’s every three months thereafter, right? Is it every – isn’t it every – is it every three or six months thereafter?” |
| “I'd first ask myself like, "Hmm, this person seems like they're high risk for HIV." And then, I would ask myself, "Do I – do I have testing that allows me to firmly confirm or deny the fact that they actually have HIV right now? […] And then, I would say to myself, like, "Okay, do I think if they like don't have a test on file or it's not that recent, are there any signs that I think that they're nonetheless actively infected with HIV and I need to test them for that before, you know, starting a conversation about preventing HIV." |
| “You’re gonna have people who are not gonna wanna come in for a HIV test every three months…I mean, that’s just gonna be a deterrent for people.” |
| **Messaging** |
| “I think it’s great in terms of like piquing interest and being quick in getting you excited about this. Um, but if it’s to help a prescriber feel comfortable prescribing, I think more detail would be helpful.” |
| **Applicability** |
| “I think you could have just like a one inspirational slide that says you may be the provider that catches someone, starts her on PrEP and prevents her from a serious infection or something. Because I think if you go about your work thinking like if I have this tool that I can bring to my patients, I may be the person that permanently improves the course of her life. Just like kind of a call to arms sort of thing I think would be nice.” |
| **Relatability** |
| “I felt like this was just like a provider like being a practitioner, a midwife, like a resident, like I didn’t – a nurse, like I didn’t feel like it was like this is like this person who the patient doesn’t feel comfortable speaking with, you know?” |
| **Design** |
| “A visual that is more like family looking and like affectionate looking, um, would again kind of just normalize that like it's – like, there's nothing wrong with you having a serodiscordant relationship, um, and that's like a normal, healthy thing.” |

**Supplement 2:**

| **Patient Animation Focus Group** |
| --- |
| **Indications** |
| “I didn’t know that – um, that Baltimore City, HIV was as high as it is. They had shared it on the news, I think like a week or two ago, and like it just kinda like caught me off-guard...” |
| **Side Effects** |
| “The stomach and the headaches, […] that’s kinda common. But like generally, kidney and bone density, that’s not like average things.” |
| “Um, you said that it doesn’t affect pregnancy or anything like that, but is there any risk – Like, if I’m on birth control, and I supplement with PrEP, is there any effect there, or they don’t affect each other whatsoever?” |
| **Access** |
| “I think the most surprising thing for me is that there is an option for people without insurance […] I don’t know how true that is. Because y’all always say that, but they’ll be, like, “Yeah. There’s an option. You can take off 10 percent.” That’s not enough.” |
| “Um, so I have a hard time seeing [...], a productive conversation about this with my gynecologist because anytime I try to be proactive or careful, I more get judgment that I’m even sexual at all.” |
| **PrEP Use** |
| “How long do it last? Is it like a shot? Well, I know it’s like a pill, but like how long do it last? Like do you have to take one every day, every week, once a month?” |
| Wait, you gotta go back every three months forever for as long as you're taking it? |
| So, if we can clear it up that – Yeah, condoms alone do prevent HIV acquisition, but it’s much more effective if you use PrEP. And if you do both together that’s even better. Um, so maybe there is a way that we could kind of, like, get that message across.” |
| **Messaging** |
| “Um, I think it was all pretty easy to understand. Because when you watch the commercials, they be using all them big words and you, like – I really don't know what they're talking about.” |
| **Use Confidence** |
| “But now, after this focus group, I’m more interested because it was kinda well-explained. I will do my own research on like the bone density and the kidneys and the side effects, but I think after this focus group that, uh, it’s something that I will have a conversation with my doctor about.” |
| “…. But, yes, I would. It’s very simple, it’s appealing. Um, if it’s 90% accurate plus on top of a condom, um, especially if you have multiple partners. Why not?” |
| **Applicability** |
| “I feel like this is a good idea for, um, you know, single women who don't intend on settling down. I feel like if you're in a, like, monogamous relationship from – like myself. I've been married for quite some time. I've been with my partner for 15 years. So, that wouldn't be something that I would do because I already gotten – like, you know, I trust the fact that he's not out here sleeping with other people and putting me at risk.” |
| **Relatability** |
| “I would say it related to me because before then, I never knew about PrEP […] And I think that it will be not only a big eye opener for me but for everyone else.” |
| “I think it's also helpful in, uh, I guess highlighting that, like – or, like, maybe dispelling some, like, misconceptions about HIV that it's – that only, like, a certain demographic can get HIV and that other people don't need to worry about it. Um, I think it's important in the beginning that it mentions that, um, heterosexual women are, like, the second most likely to contract HIV” |
| **Design** |
| The graphics – they were pretty simple. And I was – I was imagining them being animated. So, I thought this would be a little, you know, kind of funny or childish. But fairly simple. You don't want too much animation or too much color to distract from the actual, um, information. |
